# Concordance of tomographic ultrasound and multiplanar ultrasound in detecting levator ani muscle injury in patients with pelvic organ prolapse

**DOI:** 10.1371/journal.pone.0199864

**Published:** 2018-07-06

**Authors:** Weisi Lai, Lieming Wen, Yinbo Li, Xinghua Huang, Zhenzhen Qing

**Affiliations:** 1 Department of Obstetrics and Gynecology, Second Xiangya Hospital, Central South University, Changsha, Hunan, China; 2 Department of Ultrasound Diagnosis, Second Xiangya Hospital, Central South University, Changsha, Hunan, China; 3 Department of Drug Evaluation and ADR Monitoring, Food and Drug Administration, Changsha, Hunan, China; University of Florida, UNITED STATES

## Abstract

**Aim:**

To compare the evaluations of evaluate levator ani muscle injury (LAMI) by tomographic ultrasound imaging (TUI) and multiplanar (MP) ultrasound in patients with pelvic organ prolapse (POP).

**Method:**

This retrospective analysis studied women who underwent International Continence Society POP quantification examination between October 2015 and June 2016. LAMI was assessed by both TUI and MP ultrasounds. Concordance of these two testing results was analyzed. Their correlations with clinical symptoms were also studied.

**Results:**

A total of 135 women were included. All the patients with POP had a minimal LAMI depth ≥ 7 mm. Two examinations, TUI and MP, had satisfactory concordance (*k* = 0.71, *P* < 0.01). Depth of LAMI in the coronal plane demonstrated good agreement with TUI scores (*r* = 0.84; *P* < 0.01). After controlling for age, BMI, and parity, to have clinically significant POP and POP symptoms, the odds ratios (ORs) for the depth of LAMI in the coronal plane were 1.31 (95% CI 1.19–1.44) and 1.25 (95% CI 1.14–1.36), and for TUI scores were 1.72 (95% CI 1.37–2.17) and 1.63 (95% CI 1.31–2.03). Receiver operating characteristic curve analyses showed a cutoff depth of 7 mm of LAMI yielded a sensitivity of 62% and specificity of 80% for POP symptoms.

**Conclusions:**

TUI and MP had satisfactory concordance in detecting LAMI and correlated with clinical symptoms of POP.

## Introduction

Pelvic organ prolapse (POP) is a common disorder. Its causes include birth injury, chronic cough, constipation, and prior hysterectomy [1–4] Women who sustain levator ani muscle injury (LAMI) have a higher risk for POP[5,6]. A detailed evaluation of LAMI is necessary for POP management. However, it has been a challenge to perform a thorough evaluation of LAMI due to the high complexity of the pelvic floor structure and its functional anatomy [7–9].

Currently, three- and four-dimensional (3D/4D) ultrasound systems have made anatomical and functional assessment of the levator ani muscle accessible to more patients [10]. The entire levator hiatus and surrounding muscle (puborectalis or pubovisceralis muscle) can be visualized using 3D/4D translabial ultrasonography (TLUS) [11,12]. Standardized tomographic ultrasound imaging (TUI), with 8 slices obtained at 2.5 mm intervals to diagnose LAMI was first proposed by Dietz in 2007 [12]. An avulsion was defined to be positive if the plane of minimal dimension and the two slices immediately cranial exhibit abnormal insertion (i.e., discontinuity between the insertion of the muscle and the rami pubis) [12]. This methodology has been widely reported in the literature [13,14]. To obtain better understanding of LAMIs, other measurements, such as levator-urethra gap quantification [15], rendered volumes [16] and endovaginal ultrasound imaging [17], have been used to evaluate LAMI.

The ‘multiplanar’ (MP) mode, also termed ‘x-Planes’ and ‘multiplanes’, which is one of the popular display modes in 3D/4D ultrasonography, can depict cross-sectional planes through the volume in question [18,19]. For pelvic floor imaging, the MP mode could apply three orthogonal planes: midsagittal, coronal, and axial planes [20,21]. This technique could enable a multi-dimensional description of morphological characteristics of a given structure and markedly increase the utility of 3D/4D pelvic floor ultrasound [20]. The present study was designed to evaluate LAMI by both TUI and MP ultrasounds and to test their diagnostic performances in correlating with clinical POP symptoms and signs.

## Materials and methods

### Study design and selection of participants

This study was a retrospective analysis in women who visited the clinic for POP or lower urinary tract symptoms at The Second Xiangya Hospital (Changsha, China), between October 2015 and June 2016. Ethics approval for this study was granted by the Human Research Ethics Committee of The Second Xiangya Hospital on March 2017.

Inclusion criteria were, 1). Patients visited the clinics with symptoms consistent with POP or lower urinary tract symptoms; 2). Between October 2015 and June 2016; 3). Received both TUI and MP ultrasound examinations. Patients without complete clinical and sonographic data were excluded.

### Study protocol

All patients underwent a clinical interview with POP examination performed based on the Pelvic Organ Prolapse Quantification System of the International Continence Society (ICS POP-Q). Four-D TLUS was performed by a qualified sonographer. Transperineal ultrasound was performed with the patient supine after bladder emptying using a Voluson 730 or E8 system (GE Healthcare, Milwaukee, WI, USA) equipped with a 4–8-MHz curved array volume transducer that was placed on the perineum in the sagittal direction with an 85° acquisition angle. Volume acquisition was performed at rest and on maximum Valsalva maneuver with pelvic floor muscle contraction (PFMC). At least 3 Valsalva maneuvers and PFMC actions were completed per patient. Post-processing of the ultrasound volume data was performed using 4DView^TM^ version 10.3 software. All volume analyses were performed by two of the authors, who blinded to all of the clinical data. The most effective (in terms of producing organ descent) Valsalva maneuver was chosen to measure organ descent (bladder, uterus, rectal ampulla or enterocele) and levator hiatal area according to the standardized method described Dietz [20]. LAMI was assessed on the optimal PFMC volume using MP mode and standardized TUI.

POP symptoms were defined as a vaginal lump/bulge or a dragging sensation. Significant clinical prolapse was defined as POP stage 2 or higher [22,23]. Significant POP on TLUS was defined as a cystocele to at least 10 mm below the symphysis, and/or uterine descent to 15 mm above the symphysis or lower and/or rectal descent to at least 15 mm below the symphysis [23]. Significant vault prolapse was defined in patients with an enterocele to below the symphysis after hysterectomy [22, 23]. Based on a previous study by the authors involving a Chinese patient population, hiatal ballooning was defined as a hiatal area ≥ 20 cm^2^ [24].

**Fig 1:**
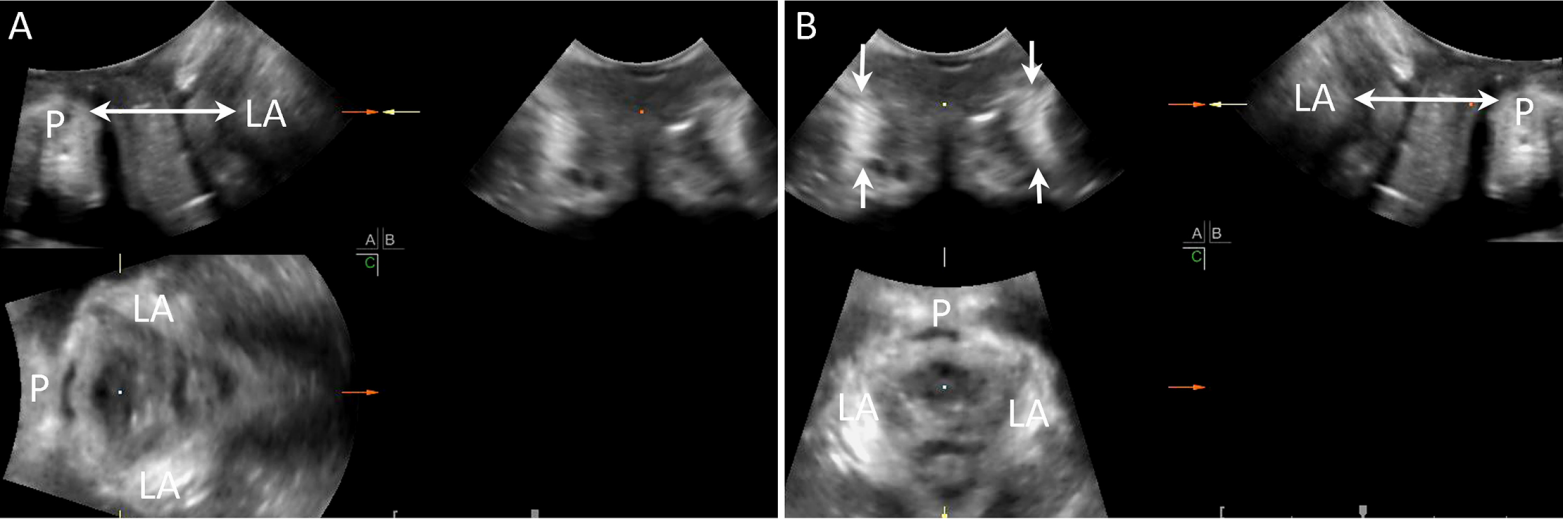
Multiplanar mode depicting three orthogonal planes of normal levator ani (LA) insertion: midsagittal plane (top left); coronal plane (top right); and axial plane (bottom left). Determination of the minimal axial plan according to anteroposterior diameter (double-sided arrow) in the midsagittal plane (top left). Image of the intact crescent-shape LA muscles (white arrows) in the coronal plane (top left). P: symphysis pubis.

The MP mode was used to depict three orthogonal planes: midsagittal; coronal; and axial (top left, top right, and bottom left of Fig 1, respectively). The minimal axial plane was determined in the midsagittal plane by the shortest anteroposterior (AP) diameter, which was the distance between the inferior-posterior aspect of pubic symphysis and the anterior border of the pubovisceral muscle, and was immediately posterior to the anorectal muscularis (Fig 1). The axial plane was rotated 90° clockwise to depict the pubic symphysis at the top, and the AP diameter in perpendicular direction; and straightly moved the dot at the center of the image, which was a marker of the structure in question, up to the level of the insertion of the levator ani muscle on the inferior rami pubis. The coronal plane of the insertion was then displayed at the top left (Fig 1), and the minimal axial plane at bottom left (Fig 1). Two crescent-shape hyperechogenic structures with an arched borderline (i.e. the levator ani muscles) were symmetrically depicted at the lateral margin of the coronal image. The hypoechogenic urethrovaginal septum or anterior vaginal wall was depicted between the crescent-shape muscles (Fig 1).

**Fig 2:**
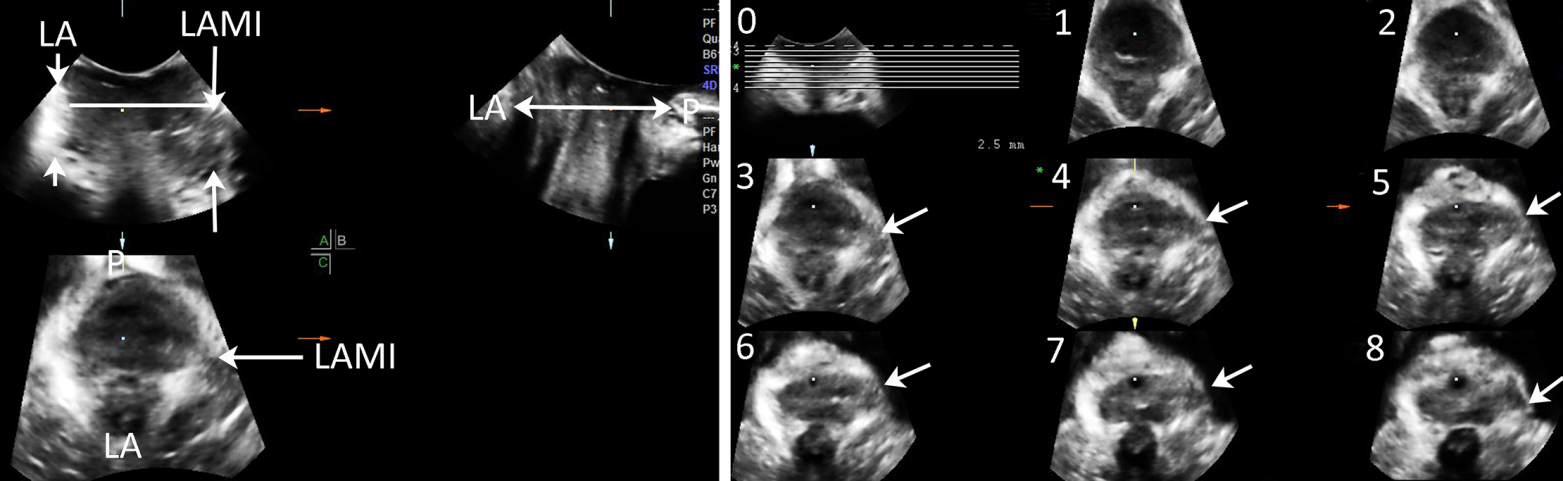
Left image depicts an intact levator ani (LA) muscle on right side (short white arrows) and a left-sided avulsion in coronal plane (long white arrows), and the measurement of the levator ani muscle injury (LAMI) depth (long white arrows) above the minimal axial plane in the coronal plane (reference line is the white horizontal line). Right image depicts levator avulsion on the left side in slices 3–8 on tomographic ultrasound imaging (TUI). TUI score of the right side is 0 and 6 on the left side. P: symphysis pubis.

A levator ani defect was defined if an irregular hypoechogenic muscle defect with discontinuous muscle borderline was detected in the crescent-shape hyperechogenic structure. The depth of the defects cranial to the minimal axial plane (i.e., the vertical length of the defect above the horizontal line, which was placed through the dot), were documented on the right and left side each (Fig 2). Of the patients with bilateral major trauma, the muscle border could not be identified. Defects were documented as ‘bilateral avulsion’ and estimated to have a depth of 12.5 mm according to a completed avulsion in 6 slices on the standardized TUI.

Levator ani trauma was also assessed using standardized TUI at slice intervals of 2.5 mm [21]. The 6 slices reaching from the plane of minimal hiatal dimension to 12.5 mm above this plane were analyzed on the right and left side each. A continuous scoring system based on tomography findings was used. Defects were scored according to the number of slices in which a discontinuity between the levator ani muscle and rami pubis was documented, using all 6 slices on each side, resulting in a maximum score of 12. Qualitative diagnostic criteria for avulsion were applied to each side separately. A complete avulsion was diagnosed if at least three central tomographic slices demonstrated an abnormal muscle insertion (Fig 2). Partial trauma was defined as < 3 abnormal planes or abnormal planes that were not consecutive [25].

Every patient received tomographic and multiplanar ultrasound examination. Presence of levator ani muscle injury was set as the main outcome of the study.

### Statistical analysis

Statistical analysis was performed using SPSS version 17.0 (IBM Corporation, Chicago, IL, USA). Cohen's Kappa test was used to examine the test agreement between TUI and MP ultrasounds. Pearson correlation analysis was applied to test the relationship between ultrasound examination and clinical symptoms. Logistic regression and receiver operating characteristic (ROC) curve analyses were used to evaluate the relationship between LAMI and POP symptoms and signs. Differences with *P* < 0.05 were considered to be statistically significant.

## Results

Of the 147 patients with clinical symptoms of POP, 12 were excluded from analysis (8 for incomplete clinical data, 4 for poor volume acquisition), leaving valid data for 135 patients. The mean (± SD) age was 48 ±10 years (range, 28–82 years), and the mean body mass index (BMI) was 22.27 kg/m^2^ (range, 16.84–31.65 kg/m^2^). The mean parity was 2 (range, 0–5), with 90% of subjects having had at least one vaginal delivery. The median age at first childbirth was 22 years (interquartile range 20–25 years). Eighty (59%) patients presented with POP symptoms. Ninety-two (69%) presented with lower urinary tract symptoms including leakage, urgency, frequency, nocturia, or voiding dysfunctionThree had undergone hysterectomy.

On clinical assessment, 33 (24%) women were POP stage 0, 23 (19%) were POP stage 1, and 79 (59%) exhibited significant POP (i.e., POP stage 2 or higher). There was a significant cystocele in 63 (47%) patients, uterine prolapse in 49 (36%), vault prolapse in 3 (2%), and rectocele in 40 (30%) patients.

On ultrasound, 83 (62%) patients exhibited significant POP, which was a cystocele in 59 (44%) patients, uterine prolapse in 51 (38%), enterocele in 4 (3%), and a rectocele in 45 (33%) patients. The mean hiatal area on Valsalva was 22.75 cm^2^, with 88 (65%) hiatal ballooning.

On the ultrasound examination, every patient with POP had a minimal depth of 7 mm for levator ani muscle avulsion. Thus, a depth of ≥ 7 mm was used to determine LAMI. Numbers of patients without LAMI, or with unilateral or bilateral avulsion, were listed in Table 1. Cohen's kappa test showed satisfactory concordance between TUI and MP ultrasound examination results (*k* = 0.71, *P* < 0.01).

**Table 1:** Discrepancies in determining an avulsion between the multiplanar mode and tomographic ultrasound imaging (TUI) (n = 135).

		Avulsion in orthogonal planes			Total	Kappa	P
		0	1	2			
Avulsion on TUI	0	83 (93.3)	11 (28.2)	0 (0)	94 (69.6)	0.71	<0.001
	1	6 (6.7)	28 (71.8)	1 (14.3)	35 (25.9)		
	2	0 (0)	0 (0)	6 (85.7)	6 (4.4)		
	Total	89 (100)	39 (100)	7 (100)	135 (100)		

Data presented as n (%) unless otherwise indicated. 0 = no avulsion; 1 = unilateral avulsion; 2 = bilateral avulsion

Logistic regression analysis was used to evaluate the association between LAMI and POP signs and symptoms, by controling age, BMI, and parity as potential confounders. In predicting clinically significant POP and POP symptoms, the odds ratios (ORs) for the depth of LAMI in the coronal plane were 1.31 (95% CI 1.19–1.44) and 1.25 (95% CI 1.14–1.36), and for TUI scores were 1.72 (95% CI 1.37–2.17) and 1.63 (95% CI 1.31–2.03).

**Fig 3:**
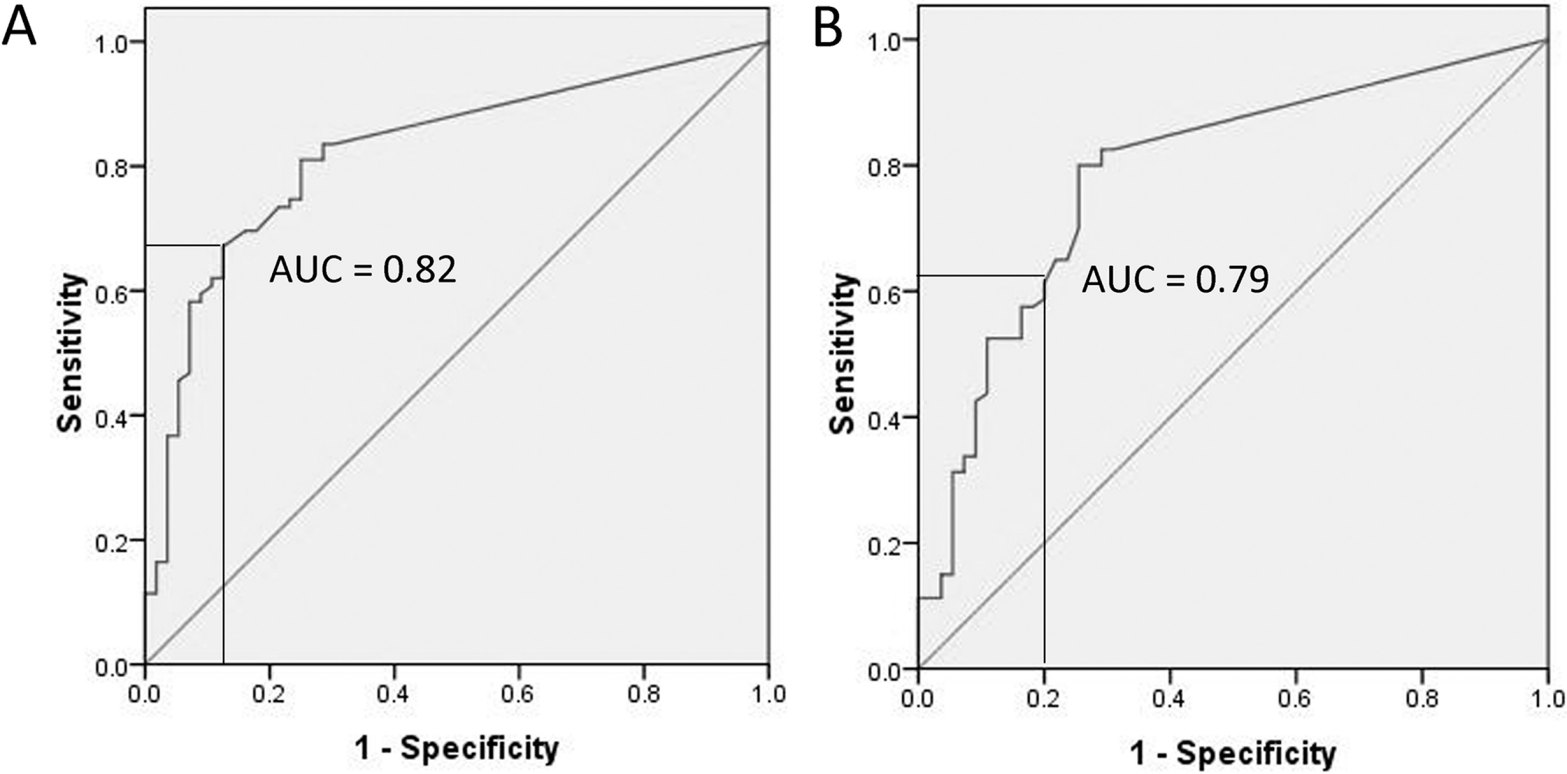
Receiver operating characteristic curve analysis demonstrating cut-offs at 7 mm for levator ani muscle injury (LAMI) depth against International Continence Society pelvic organ prolapse (POP) quantification (ICS POP-Q) stage 2 or higher (A), and prolapse symptoms (B).

**Fig 4:**
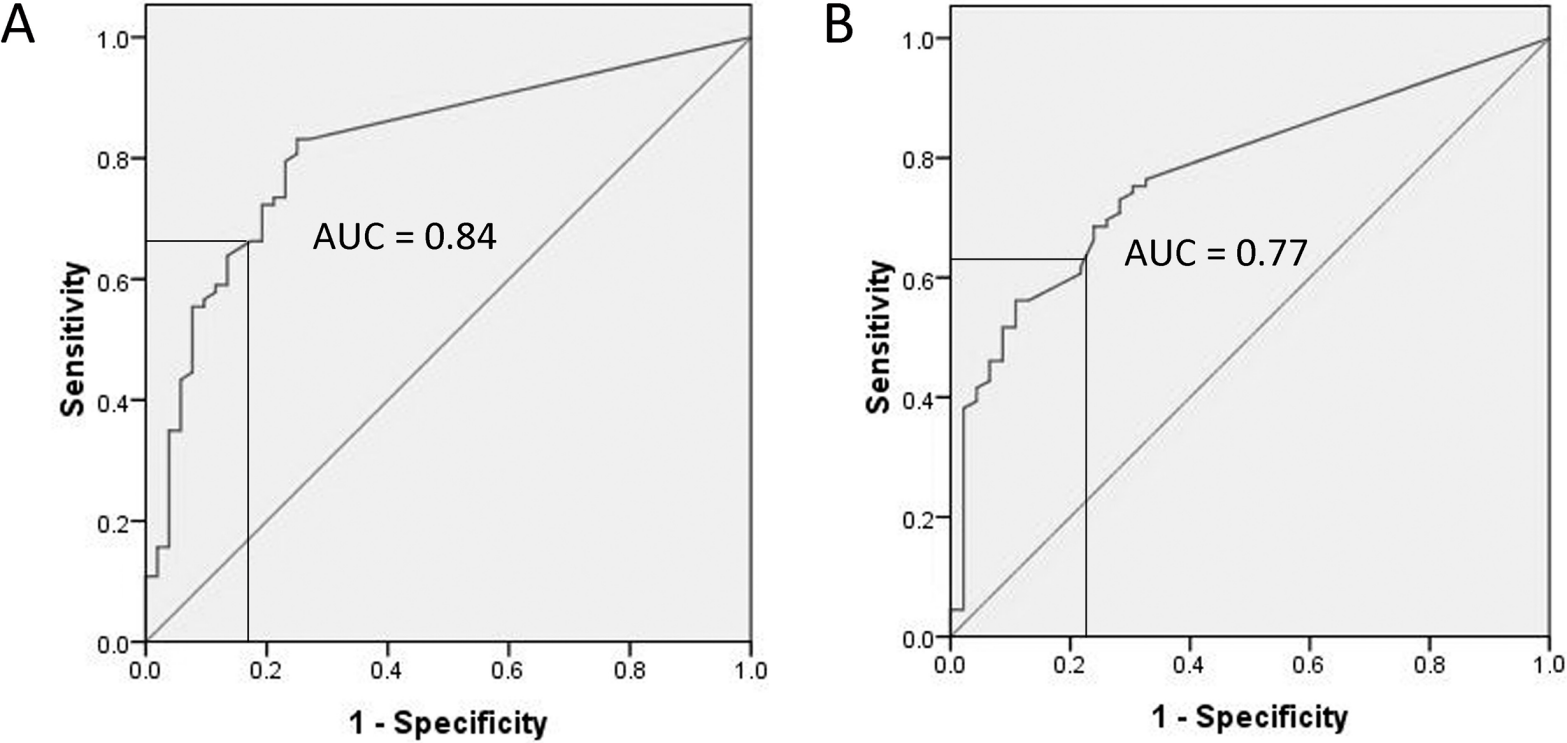
Receiver operating characteristic curve analysis demonstrating cut-offs at 7 mm for levator ani muscle injury (LAMI) depth according to significant pelvic organ prolapse on translabial ultrasonography (TLUS) (A) and hiatal ballooning (B).

ROC curve analysis was used to test the diagnostic performance of LAMI depthThe AUCs for the depth of LAMI were 0.82 (95% CI 0.75–0.90), 0.79 (95% CI 0.71–0.87), 0.84 (95% CI 0.75–0.89) and 0.77 (95% CI 0.69–0.85) against POP stage 2 or higher, POP symptoms, significant POP on TLUS and hiatal ballooning (Figs 3 and 4). A cutoff LAMI depth of 7.0 mm yielded a sensitivity of 67% and specificity of 88% against POP stage 2 or higher, and a sensitivity of 62% and specificity of 80% against POP symptoms (Fig 3). This cutoff produced a sensitivity of 64% and specificity of 83%, and a sensitivity of 62% and specificity of 78%, according to significant POP on TLUS and hiatal ballooning, respectively (Fig 4).

## Discussion

The most common morphological abnormality of the levator ani is a unilateral avulsion of the pubovisceral muscle off the pelvic sidewall [26,27], which is a significant risk factor for POP recurrence after surgery [5,6,28]. Many studies have reported that mesh implantation may improve the condition [28,29]. Levator ani muscle repair is one of several surgical treatments for POP in women with levator avulsion [30]. Clearly, LAMI assessment is absolutely necessary for better clinical consultation and surgical planning.

TUI with 3D/4D TLUS is currently the most popular validated method to detect LAMI [9,13,14]. The coronal plane of the insertion of levator ani muscle on rami pubis, which was the reference plane in standardized TUI, is shown at the left top corner on TUI (Fig 2). The extent of LAMI was measured according to the number of slices with a fixed interval of 2.5 mm [12,25]. It is plausible that measurements of the depth of LAMI in the coronal plane were consistent with levator ani trauma scores on TUI. In our study, the reproducibility of detecting LAMI using 3D/4D TLUS was evident due to the good consistency (*r* = 0.84) between LAMI depth and TUI scores, as well as limited discrepancy (k = 0.71) in determining levator ani muscle avulsion between the MP mode and TUI.

MP mode enables clinicians to obtain a more accurate evaluation of LAMI using multidimensional visual assessment as well as a numerical data. The midsagittal plane displayed the slice position, which was at the anterior vaginal wall or urethrovaginal septum; the axial plane depicted the morphological characteristics of the levator ani muscle, which encloses the minimal levator hiatus; and the coronal plane revealed the range of LAMI at the insertion. The configuration and limits of LAMI were accurately presented in the orthogonal planes. It appears possible to improve the evaluation of LAMI in orthogonal planes. Regression analysis revealed that LAMI was an independent risk factor for significant POP (OR = 1.31), and ‘major trauma’ (i.e., LAMI depth ≥ 7 mm) was strongly correlated with POP symptoms and signs (OR = 6.13 and 4.88). LAMI in the coronal plane demonstrated excellent performance in predicting significant POP.

While our results confirmed the validity of this technique based on clinical and research practice, there were several limitations to this study that should be acknowledged. First, a single slice in the coronal plane cannot account for the entire insertion of levator ani muscle on the inferior pubic ramus. It is possible that our measurements were not exactly performed on the largest LAMI. Second, the volume analysis was performed by experienced operators to ensure the reliability of the measurements. However, the repeatability and practicability of this method need to be tested in further studies.

## Conclusions

TUI and MP had satisfactory concordance in detecting LAMI and correlated with clinical symptoms of POP. A LAMI with depth ≥ 7mm might be used as the cutoff to determine POP.

## Supporting information

S1 Data Raw data. (XLS)
